# Born at the Wrong Time: Selection Bias in the NHL Draft

**DOI:** 10.1371/journal.pone.0057753

**Published:** 2013-02-27

**Authors:** Robert O. Deaner, Aaron Lowen, Stephen Cobley

**Affiliations:** 1 Department of Psychology, Grand Valley State University, Allendale, Michigan, United States of America; 2 Department of Economics, Grand Valley State University, Grand Rapids, Michigan, United States of America; 3 Faculty of Health Sciences, University of Sydney, Sydney, New South Wales, Australia; University of Minnesota, United States of America

## Abstract

Relative age effects (RAEs) occur when those who are relatively older for their age group are more likely to succeed. RAEs occur reliably in some educational and athletic contexts, yet the causal mechanisms remain unclear. Here we provide the first direct test of one mechanism, selection bias, which can be defined as evaluators granting fewer opportunities to relatively younger individuals than is warranted by their latent ability. Because RAEs are well-established in hockey, we analyzed National Hockey League (NHL) drafts from 1980 to 2006. Compared to those born in the first quarter (i.e., January–March), those born in the third and fourth quarters were drafted more than 40 slots later than their productivity warranted, and they were roughly twice as likely to reach career benchmarks, such as 400 games played or 200 points scored. This selection bias in drafting did not decrease over time, apparently continues to occur, and reduces the playing opportunities of relatively younger players. This bias is remarkable because it is exhibited by professional decision makers evaluating adults in a context where RAEs have been widely publicized. Thus, selection bias based on relative age may be pervasive.

## Introduction

Relative age effects (RAEs) occur when those who are relatively older for their age group are more likely to succeed. RAEs are perhaps most often associated with Canadian ice hockey, where nearly 40% of players on elite junior teams are born in the first quarter of the year, meaning that, because of a January 1 cut-off date, they would have been consistently older than their age group peers [1]–[5]. RAEs have been described in numerous athletic and educational contexts, and there is consensus that RAEs are unfair to relatively younger individuals and that the implied loss of talent may negatively impact societies [6]–[14] (but see [15], [16]).

Eliminating RAEs has proven challenging, however, in part because of difficulties in identifying which of the plausible mechanisms are causal [7], [10], [11], [13], [17]. For example, all else being equal, the youngest boy on a youth hockey team will be smaller, weaker, less emotionally mature, and less skillful; these disadvantages may lead to other ones, including diminished confidence, less instruction from coaches, reduced likelihood of being selected for elite teams, and, ultimately, a greater likelihood of dropping out of the sport. Thus, there are many potentially causal mechanisms, and they are likely to interact.

Here, we attempt to isolate and demonstrate the occurrence of one mechanism: selection bias. By selection bias, we mean that evaluators (e.g., teachers, coaches) mistakenly grant fewer opportunities (e.g., instruction, access to elite group or team) to relatively younger individuals than is warranted by their latent ability or talent. Although selection bias is widely accepted as a contributor to RAEs [7], [10], [11], [13], [17], there is little direct evidence for it (but see [10], [11]), apparently owing to the difficulty of obtaining measures of both perceived and latent talent. Furthermore, in most studies consistent with selection bias, the selector's preference for relatively older individuals might be rational, given the selector's aims and the greater maturity of relatively older individuals. For example, a coach selecting a youth team may favor relatively older players because the coach's performance may be evaluated based on her team's success; similarly, a teacher may select mostly relatively older pupils for a gifted academic program because they are most likely to benefit from and contribute to it.

We tested the hypothesis of selection bias by evaluating entry drafts of the National Hockey League (NHL), the world's premier professional ice hockey league. NHL teams generally draft players once they have reached 18 years of age [18], and teams select players in order of their perceived talent [19], [20]. Nevertheless, there is uncertainty regarding the career trajectory of players, and nearly half never develop sufficiently to play a single game in the NHL. The logic of our study is that a player's draft slot serves as a measure of their perceived talent whereas career productivity indicates their realized talent. If selection bias occurs, then, for any given draft slot, relatively younger players will enjoy more productive careers.

The possibility of selection bias in NHL drafting was suggested by a recent study [3]. It reported that 40% of Canadian-born first round NHL draft selections from 2007–2010 (n = 62) were born in the first quarter, but only 28% of Canadian-born NHL players were born in the first quarter. Moreover, in a large sample (n = 1,003), first quarter born players comprised fewer than 25% of Canadian All-Star and Olympic team members and had substantially shorter NHL careers than players born later in the year.

We expanded on this study in several ways. First, our analyses of productivity included all selections from all draft rounds for a period of 27 years, meaning that our sample size of draftees was 44 times larger (n = 2,736 Canadian-born non-goaltenders). Second, we used analytic methods to directly test for selection bias while controlling for potentially confounding variables. Most crucially, we tested whether birth quarter was associated with productivity once draft slot was controlled; this is vital because relatively younger players may be drafted earlier [21]. Third, we investigated a potential mediator of selection bias, the decision to become draft eligible. Fourth, we tested for changes in selection bias over time. Finally, we examined whether selection bias reduces relatively younger individuals' playing opportunities.

## Results

**Figure 1:**
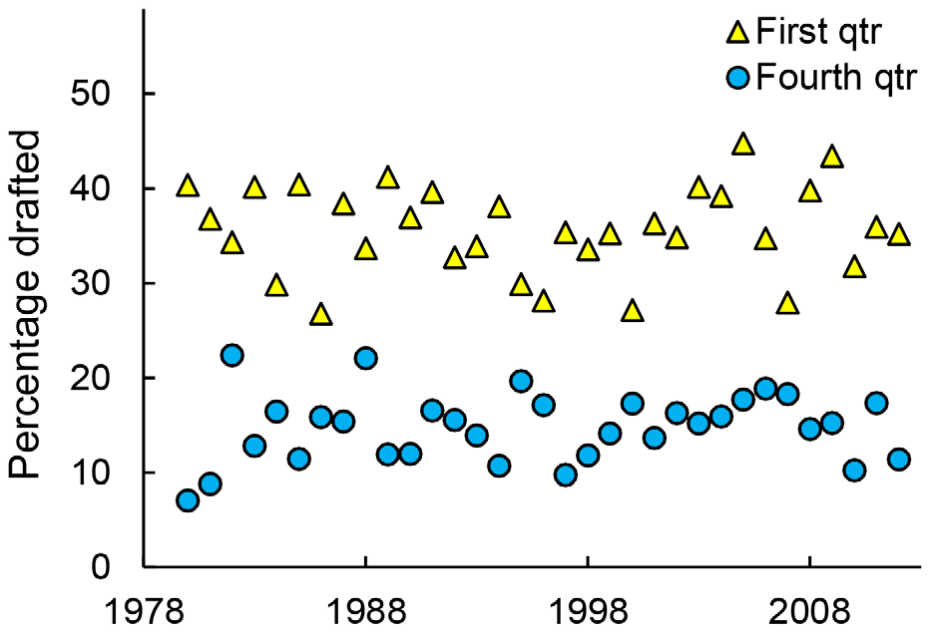
Percentage of NHL draftees born in the first or fourth quarter over time. Blue circles indicate first quarter; yellow triangles indicate fourth quarter.

We began by confirming previous studies showing that the number of players drafted differed by birth quarter [3], [21]. Overall, 36% of draftees came from the first quarter and 14.5% came from the fourth quarter. As shown in Figure 1, this discrepancy occurred in every draft from 1980 to 2012, and there was no indication of temporal change for the percentage of fourth quarter players drafted, the percentage of first quarter players drafted or their difference (all ps>.45).

### Productivity measures

In assessing NHL productivity and selection bias (i.e., all analyses below), we only considered players drafted prior to 2007 because little data have accrued for players drafted later than this. As our primary productivity measure, we used career games played [3], [19], [22], [23] (see also [24], [25]). The logic is that if a team decides to utilize a player, they must view him as likely to contribute more than their alternatives. Games played is advantageous because it accommodates various positions and roles (e.g., defenseman, enforcer, scorer, checking forward). Furthermore, supporting its validity, games played correlated with three other *prima facie* indicators of productivity, goals per game (r(1459) = 0.46, p<.0001), assists per game (r(1459) = 0.57, p<.0001), and points (i.e., goals plus assists) per game (r(1459) = 0.57, p<.0001)(see [22]). Games played also correlated with a productivity measure that encompasses offensive and defensive contributions, plus-minus per game (r(1459) = 0.23, p<.0001); plus-minus is goals scored by own team while playing less goals scored against own team while playing. For both defensemen and forwards, games played correlated with all four measures of productivity (all ps<.0001). As a secondary productivity measure we used career points scored.

### Selection bias

**Figure 2:**
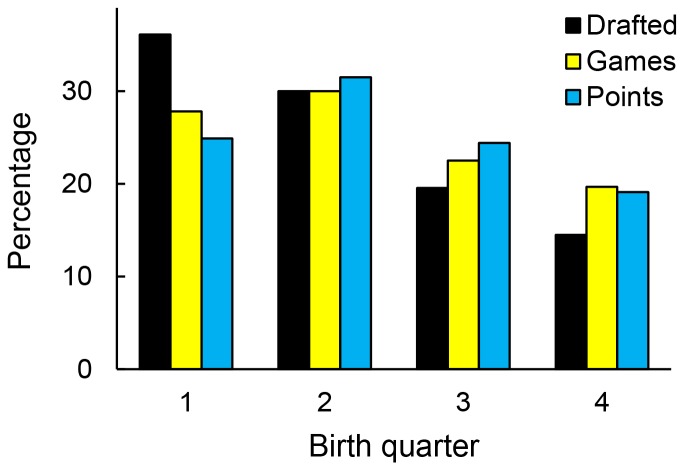
Percentage of draftees and productivity by relative age.

Supporting the hypothesis of selection bias, the percentage of total productivity achieved by players born in the third and fourth quarters was far greater than the percentage of players drafted from these quarters (Fig. 2). For example, although 14.5% of draftees were born in the fourth quarter, these individuals played 20% of the games (477,000) and scored 19% of the points (209,000) accumulated by draftees in our sample. By contrast, those born in the first quarter dramatically under-produced, given that they constituted 36% of draftees.

That relatively younger players are relatively more productive (Fig. 2) is consistent with the selection bias hypothesis but does not unambiguously support it. The reason is that players drafted earlier are expected to be more productive, and relatively younger players might be drafted earlier. This pattern was reported previously [21], and we also found it here with a larger sample (ordinary least squares regression: R^2^ = .01, F(3,2732) = 9.29; p<.0001; mean draft slot Q1 = 119; Q2 = 114; Q3 = 124; Q4 = 99). The critical question, therefore, is whether birth quarter is related to productivity when draft slot is controlled.

We addressed this question using Tobit regressions to account for players who never reached the NHL. Indicator variables were included to estimate the effect of later birth quarters (i.e., second, third, fourth) compared to the first quarter, and these models also controlled for draft slot, position (i.e., defenseman, forward), height, and year of draft. The results indicated that, compared to first quarter draftees, relatively younger draftees played more games (Q2 = 71 games, p = .005; Q3 = 160, p<.001; Q4 = 162; p<.001) and scored more points (Q2 = 52 points, p = .002; Q3 = 110, p<.001; Q4 = 88; p<.001).

A key question is whether selection bias applies even to early draft selections, where decisions are weighed more carefully [19]. We thus repeated these regression analyses restricting our analyses to players selected in the first 30 slots. This currently corresponds to the first round of the NHL draft (i.e., each team makes their first selection), although it represented almost 1.5 rounds at the beginning of our study period when there were 21 teams. The magnitude of selection bias was similar to when we included all draftees (above), although owing to small samples, the coefficient estimates were not always significant (games: Q2 = 80, p = .11; Q3 = 223, p<.001; Q4 = 92, p = .09; points: Q2 = 69, p = .08; Q3 = 162, p<.001; Q4 = 45, p = .30).

**Figure 3:**
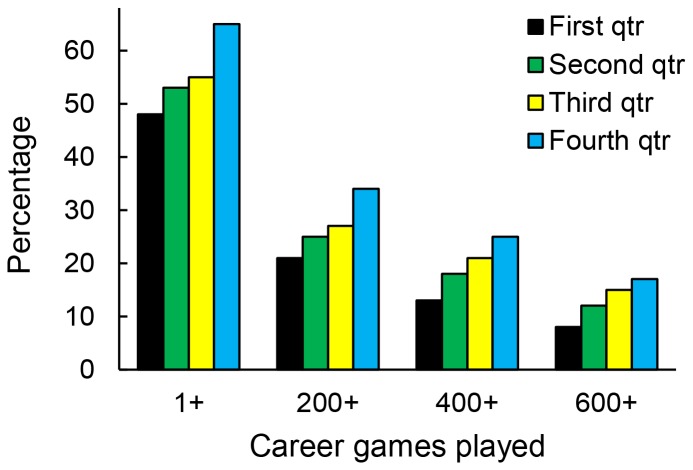
Percentage of draftees from each birth quarter achieving benchmarks of career games played.

The magnitude of selection bias can be appreciated by examining the percentage of players from each birth quarter reaching major career benchmarks [19] (Fig. 3; Table 1). The differences are striking: across the sample (n = 2,736), 13% of those born in the first quarter reached 400 games, whereas the values for the other quarters were, respectively, 18%, 21%, and 25% (Odds-ratio [OR] compared to first quarter: 1.47, 1.78, 2.23). Furthermore, the percentages from each quarter reaching 200 points were 8%, 13%, 15%, and 17% (OR: 1.71, 2.02, 2.36). Although selection bias is also manifest in the basic benchmark of playing at least one NHL game, the effect was more modest; the percentages were 48%, 53%, 55%, and 65% (OR: 1.22, 1.32, 2.01). Because the majority of more highly drafted players (i.e., selected in first 100 slots), reached this benchmark, the effect was driven by players drafted later (Table 1).

**Table 1:** Percentage of draftees achieving career benchmarks by relative age and draft slot.

		Games				Points	
Birth quarter	n	1+	200+	400+	600+	100+	200+
Draft slots 1–100							
1	444	73	34	22	14	23	13
2	400	74	40	29	20	31	22
3	231	75	48	39	28	40	28
4	225	81	48	37	25	40	26
Total	1300	75	41^*^	30^*^	20^**^	31^**^	21^**^
Draft slots 101+							
1	541	28	10	6	4	6	3
2	420	34	11	7	4	6	5
3	304	40	12	8	5	7	6
4	171	44	16	9	6	9	6
Total	1436	34^*^	11	7	4	7	4
All draft slots							
1	985	48	21	13	8	14	8
2	820	53	25	18	12	18	13
3	535	55	27	21	15	22	15
4	396	65	34	25	17	27	17
Total	2736	53^*^	25^**^	18^**^	12^**^	18^**^	12^**^

*Note*. Chi-square tests of independence were used to test if frequency of achieving benchmark was dependent on birth quarter. ^*^ p<.01; ^**^ p<.001.

Although relatively younger players (specifically those born in the fourth quarter) were drafted somewhat earlier than those born in the first quarter, the regressions controlling for draft slot indicate that, given their productivity, they were not drafted early enough. To further illustrate this, we estimated the discrepancy in drafts slots for a first quarter born player and relatively younger player who had played the same number of games. As above, the coefficients on the birth quarter dummy variables (i.e., intercepts) were all significantly different from the first quarter (all ps<0.01), and there was no indication that the slopes differed (F(3, 2492) = 0.58, p = .63; all individual slopes ps>.20). The regressions indicated that a second quarter draftee played the same number of games as a first quarter one if drafted 20 slots later; the discrepancies for the third and fourth quarters were 45 and 43 slots.

### Temporal change

Systematic biases in some professional sports drafts have diminished over time [24], [26].

To test for temporal change in selection bias in the NHL draft, we used the draft slot discrepancy measures described in the previous paragraph. Because the effects were large, we focused on the first and third quarter discrepancy and the first and fourth quarter discrepancy. Due to the modest sample for each year, we calculated discrepancies with a five year moving window.

**Figure 4:**
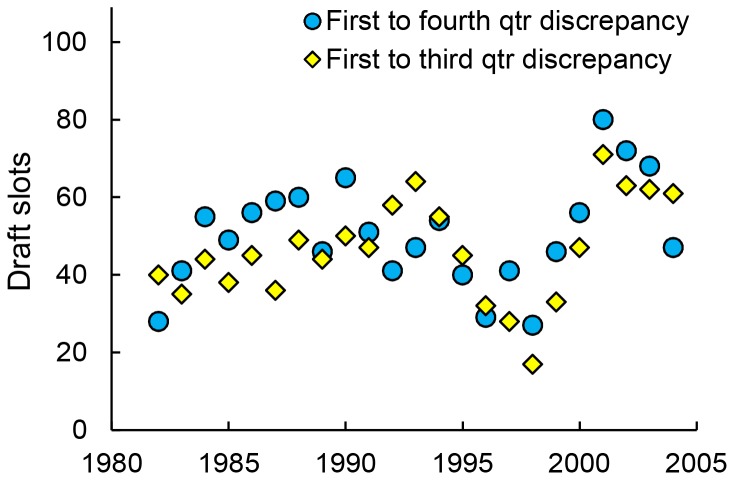
Selection bias against relatively younger draftees over time. Draft slots indicate the draft selection discrepancy between individuals born in the first quarter and later quarters with equivalent career games played. Yellow diamonds indicate first to third quarter discrepancy; blue circles indicate first to fourth quarter discrepancy.

Figure 4 shows that both draft slot discrepancies were in the predicted direction for all 23 of these five year epochs. Both discrepancies were significant (p<.05) for 20 of 23 epochs. The first to third discrepancy did not reach significance at epochs with midpoints of 1987, 1997, and 1999; the first to fourth discrepancy was not significant for 1982, 1996, and 1998. Both discrepancies tended to increase over time, although in neither case was this change significant (first to third: r(21) = .33, p = .13; first to fourth: r(21) = .25, p = .26). Thus, we found no indication that selection bias based on relative age decreased over time.

### Decision to become draft eligible

We next considered a potential mediator of selection bias, the player's decision to become draft eligible. In particular, prior to 2005 players were not uniformly draft eligible based on their birthdays and had to inform the NHL office that they wished to ‘opt in’ to the draft [18], [27]. Unfortunately, we were unable to obtain data regarding actual opt in decisions. However, based on birthdays and year of draft selection, we determined that 70% of players were drafted in their first year of potential eligibility, meaning that at least this percentage opted in. Moreover, players drafted in their first year of eligibility were drafted substantially earlier than those drafted in their second or third year of eligibility (first mean = 99; later mean = 152, t(2734) = 17.5, p<.0001). This suggests that many players who were not drafted in their first year of eligibility had previously opted in but had not been drafted due to their perceived lesser ability. Nonetheless, in the 2005 and 2006 drafts, when all players were automatically eligible, 86% were drafted in their first year of eligibility whereas only 67% were drafted in their first year of eligibility in the 2003 and 2004 (opt in) drafts. This indicates that an appreciable number of players did not opt in prior to 2005.

To address the opt in decision as a potential mediator, we first examined whether there is an effect of year of potential draft eligibility on productivity. We found evidence for such an effect: players drafted in their second or third year of potential eligibility were substantially more productive than those drafted in their first year of potential eligibility; this held in Tobit regressions controlling for draft slot, position, height, and year of draft (games = 80, p = .001; points = 55, p = .001).

However, if the opt in decision was truly a mediator, then the relation between birth quarter and productivity should disappear or significantly weaken when regressions control for the year of potential draft eligibility. Contrary to this, birth quarter remained a strong predictor of productivity when year of potential draft eligibility was added to the regressions, which also included draft slot, position, height, and year of draft. This was true for games (Q2 = 69, p = .008; Q3 = 158, p<.001; Q4 = 165; p<.001) and points (Q2 = 49, p = .004; Q3 = 108, p<.001; Q4 = 91; p<.001). Crucially, the interactions between birth quarter and year of potential eligibility (i.e., first, or later) were not significant in any case (all ps >.10). Furthermore, when we repeated our original tests of selection bias using only the 70% of players drafted in their first year of potential eligibility, we still found that players born in the second, third, and fourth quarters were far more productive than those born in the first quarter (games: Q2 = 64, p = .03; Q3 = 139, p<.001; Q4 = 134; p<.001; points: Q2 = 47, p = .02; Q3 = 99, p<.001; Q4 = 79; p = .001). We found similar results when we only included the 30% of players drafted in their second or third year of eligibility (games: Q2 = 78, p = .10; Q3 = 191, p<.001; Q4 = 243; p<.001; points: Q2 = 58, p = .08; Q3 = 131, p<.001; Q4 = 119; p = .01). Thus, there is no indication that the opt in decision mediated selection bias based on relative age.

### Costs of selection bias

Finally, we explored a potential cost of selection bias, namely that later draft selections may receive fewer playing opportunities than their latent talent would warrant. The reason is that when a player's ability to make a net contribution to their team is ambiguous, teams will more often grant the benefit of doubt to a player to whom they have invested more highly (i.e., drafted earlier). This tendency is called ‘escalation’ in studies of professional basketball [28], [29].

The hockey data yielded evidence consistent with escalation: games played was jointly predicted by points per game and draft slot; crucially the draft slot regression coefficient was negative (R^2^ = .50, F(2,1456) = 736.8, p<.0001; β_draft slot_ = −.69, p<.001; β_points per game_ = 7.26, p<.001). Furthermore, when position, height, and plus-minus per game were added as predictors, the effect of draft slot remained substantial (R^2^ = .51, F(5,1275) = 262.4, p<.0001; β_draft slot_ = −.69, p<.001; β_points per game_ = 7.22, p<.001; β_position-forward_ = −76.1, p<.001; β_plus-minus_ = −.26, p = .10; β_height_ = −1.9, p = .63). Thus, a player who is drafted ‘later than they should be,’ must produce more to receive the same playing opportunities.

## Discussion

Here, we have demonstrated that, for 27 years, relatively younger NHL draftees have enjoyed substantially more productive careers than would be predicted by their draft slots. Moreover, the pattern of drafting far fewer relatively younger individuals has not waned and has occurred again in recent years, including in the 2012 draft (Fig. 1). Before we explore the implications of this selection bias, we must address three questions.

First, are our findings novel, given earlier studies? It was previously known that the unequal distribution of players by birth quarter is greater in junior hockey than in the NHL [2], [3], [5]. This pattern is consistent with selection bias but does not require it. This is because relatively younger players in junior hockey might, on average, be more talented than relatively older players, and NHL teams might recognize this and draft accordingly. In fact, a previous study reported that relatively younger players were drafted earlier [21], and we confirmed the effect with a larger sample. Thus, if NHL teams simply drafted based on (unbiased) perceptions of talent, there would be no relationship between relative age and productivity. We showed, however, that there is a strong relationship, even when draft slot is controlled.

Our findings are also novel in that they apparently constitute the first demonstration that, in sports, selection based on relative age can be irrational (see below). As noted in the Introduction, although many studies show that relatively older players are more likely to be selected for youth elite teams, none of these studies address whether these selection patterns are disadvantageous for the teams and selectors given that relatively older players generally have greater ability at the time of selection. Because we had good measures of both perceived talent (i.e., draft slot) and realized talent (i.e., career productivity), we were able to address this question directly.

A second question is whether there could there be some rational basis for teams drafting relatively younger individuals later than is warranted by their career productivity. A study of the Australian Football League draft illustrates how this might occur: indigenous players significantly outperformed their draft slots; however, the authors could not rule out that this selection bias might be rational because the recruitment and retention of indigenous players might require additional resources, such as assistance with housing and cultural acclimatization [25]. Similarly, a study found that South American players in the Premier division of the English professional soccer league tended to be overpaid in terms of their effects on team performance; however, this preference for South American players might be rational because the presence of these players, perhaps owing to their stylish play, was associated with increased attendance [30].

Although an explanation analogous to one of these examples cannot be logically excluded, it is difficult to conceive of a reasonable one. For example, most fans are apparently unaware of players' birthdays and do not have stereotypical beliefs about the playing style or abilities of players based on relative age. Furthermore, as we showed, the selection bias was robust to different measures of productivity and was undiminished when possible confounds were incorporated. Perhaps the most plausible idea is that relatively older individuals are rationally preferred, despite their worse long-term productivity, because they are more likely to make an early contribution to a team. In particular, a relatively older player would be absolutely older than a relatively younger one, meaning that they would be further along in their development.

Contrary to such reasoning, however, draft eligibility rules dictate that players born from January 1 to September 15 of year X first potentially become draft-eligible at the same time as players born September 16 to December 31 of year X-1 [18], [19]. Paradoxically, then, relatively older individuals (e.g., born in first quarter) are absolutely younger than those in their draft-eligible cohort who were born in the fourth quarter. Furthermore, our results show that relatively older individuals are not only less likely to reach long-term career benchmarks (i.e., 400 career games); they are also less likely to even play a single game (Fig. 3; Table 1). Thus, it is reasonable to conclude that the selection bias we have shown is not in the drafting teams' best interest (i.e., it is irrational).

A third question is whether there is in fact an RAE in the NHL. Representation and productivity in the NHL differed substantially by birth quarter (Fig. 2; Table 1). Nonetheless, one might argue that because the RAE is smaller in the NHL than in junior leagues [2], [3], [5] there is no mechanism actively contributing to the RAE in the NHL [3] (see also [15], [31]–[33]). In other words, the differential representation by quarter in the NHL is a legacy of past RAE mechanisms. Our demonstration that relatively younger draftees typically achieve greater careers certainly supports this view. Indeed, one might suggest that ‘the cream rises to the top,’ and, despite where they are drafted, truly talented players, whether relatively older or younger, will manage to develop their abilities and achieve long and productive careers [3], [34], [35].

This argument has some validity, yet it overlooks that being drafted ‘later than one should,’ or not being drafted at all, can be costly. Our analysis of escalation effects shows that, once in the league, players who are drafted earlier receive greater playing opportunities than is warranted by their productivity. These opportunities should allow them to more easily establish their reputations, and these opportunities can translate directly into compensation because recently drafted players can earn most of their income through playing-dependent performance bonuses [18]. Moreover, prior to the collective bargaining agreement of 1995, rookie salaries were far higher for players drafted earlier [36]. Indeed, the likely benefits of early selection are revealed in the behavior of potential draftees: they voluntarily endure days of draft evaluation sessions (e.g., interviews, physiological testing) to improve their draft prospects [19]. Thus, if there was no selection bias against relatively younger individuals, there would be more of them in the NHL, and they would produce and earn more.

Our demonstration of selection bias against relatively younger draftees is important because it suggests that selection bias may occur broadly. Obviously, selecting professional hockey players differs considerably from selection in other contexts, yet we believe that, all else being equal, selection bias could be at least as great elsewhere.

Consider NHL teams' draftee evaluations compared to German teachers' recommendations about their 10 year-old pupils; these recommendations concerning the suitability of secondary school tracks (e.g., basic or academic) are substantially biased against relatively younger children and have life-long effects [11], [12].

- NHL teams are evaluating 18 year-olds, not 10 year-olds, meaning that any age gap is proportionally almost twice as large [13]. In fact, most discussions of RAEs state or imply that, although the consequences of RAE mechanisms may persist into adulthood, most mechanisms will be active in children or early adolescents [3] (see also [15], [31]–[33]).
- NHL drafting is done by scouts and general managers who are highly motivated to make accurate evaluations due to the high-stakes involved and the scrutiny given to their decisions [19], [20], [24], [26]. By contrast, teachers making recommendations may have less information, less time for deliberation, and may face few or no consequences for poor decisions.
- NHL teams are probably aware of RAEs. RAEs in hockey have been documented in the academic literature since the mid-1980s [2], [4], recognized as potentially important by national federations since the mid-1990s [5], and popularized in a bestselling book [37]. In fact, hockey scouts' evaluations frequently consider the relative age of the scouted player compared to their teammates and opponents [19]. Teachers, on the other hand, may have little or no awareness of RAEs [8], [11].

These points collectively indicate that selection bias may be a pervasive contributor to RAEs and that merely instructing selectors to account for it may be ineffective (see [8], [11]).

A final question is what factor(s) causes NHL teams to underestimate the future productivity of relatively younger players. The obvious candidate is maturity-related differences in physiological (e.g., power) and technical characteristics [38]. This possibility might seem unlikely because, as noted above, individuals born in the fourth quarter are, in most cases, absolutely older than others in their draft-eligible cohort [18], [19]. However, player evaluations are based on several years of consideration and, once a player is initially evaluated by professional scouts, perhaps in their early teens, this can, in various ways, affect future evaluations. For instance, relatively younger players may be less likely to be selected for the most elite junior teams [19], [20] (see also [1]–[5]); level of competition, has, in turn, been shown to systematically bias drafting in professional sports [39]. Similarly, prior to 2005, players' decisions regarding what year they ‘opted in’ to the draft might have mediated the selection bias; however, our analyses found no evidence for this.

Another possibility is that selection bias in NHL drafting is based on some sort of ‘underdog’ effect whereby relatively younger individuals, because they have faced greater social challenges [9], [14] (see also [34]), compensate by developing greater adaptability to different roles or better work habits; these traits then lead to greater long-term achievement [3], [32] (see also [40]). An underdog effect would have interesting implications, but there is little direct evidence for it yet. Furthermore, even if an underdog effect occurs, its importance is generally countervailed by other mechanisms that typically make being relatively younger disadvantageous [6]–[14].

## Materials and Methods

We obtained data from the official National Hockey League web site, www.nhl.com. In cases where data were unavailable, we attempted to obtain them from another site, www.hockeydb.com/. We recorded information on draft year, draft slot, player name, birth country, birth month, birth year, and whether the player was a goalie, defenseman, or forward (i.e., center, wing). We initially gathered data on all 8,186 draft selections made from 1980–2012. We did not consider data prior to 1980 because, before this, RAEs did not occur consistently [41], [42], and birth dates for draftees who did not reach the NHL were sparse. For analyses involving productivity, we excluded players drafted from 2007–2012; we did this because these players have had little opportunity to accrue production.

The number of selections across our sample varied across years from 210 to 293. This variation was due to an increase in the number of NHL teams (from 21 in 1980 to 30 since 2000) and a decrease in the number of draft rounds (from 12 during the most drafts in the 1980s to 7 since 2005). In less than 1% of cases, a player was drafted, did not sign a contract, and then was selected a second time in a later draft; we excluded players' data from their second drafting.

For several reasons, we excluded those who were not Canadian-born (55% of draftees). First, draft eligibility rules differ for individuals playing outside of North America [18]. Second, European based players often play in their domestic leagues or have national service obligations; these can raise obstacles to their playing in the NHL and can affect the willingness of teams to draft them [19]. Third, because there are far more Canadians than any other nationality in the NHL and the Canadian developmental system is comparatively homogenous, most previous RAE studies focused on Canadian-born players [2]–[5], [41]. We also excluded goalies. We did this because they constitute a small percentage of draftees (10%), and the development and evaluation of goalies differs substantially from that of forwards and defensemen [19], [20]. Our preliminary analyses yielded no indication of selection bias for goalies, using games as the productivity measure (for each later birth quarter, p>.70; combined F-test, p = .95). If we had included goalies, the selection bias shown in our Results would have been highly similar (for each later quarter, ps<.01).

Because players have unequal opportunities to participate in the playoffs, we considered only regular season production. Teams played 80–84 games per annual regular season during the sample, with the exception of one abbreviated (1994–1995) and one cancelled season (2004–2005) [19], [36]. Our productivity measures were based on career totals at the conclusion of the 2011–2012 season. Because many players have not yet completed their careers, they will continue to accrue productivity. Although this means that draft year will correlate with productivity, there is no known reason to expect that this would spuriously produce evidence of selection bias.

Because roughly 45% of draftees never play in a single NHL game, productivity measures have a non-normal distribution. Thus, when predicting productivity, we used censored regression models (i.e., Tobits). When conducting multiple regressions to address escalation effects, we reported regression coefficients that were not standardized, so that the coefficients represent conditional marginal effects. For example, the first set of regression results indicate that, when draft slot is fixed, games played increases by 7.24 for each additional point per game scored. All analyses were conducted using two-tailed statistical tests with Stata v.12.1 (College Station, TX).
